# Assessing the effects of menopause and obesity on matriptase-2 and neurokinin B concentrations: A cross-sectional study

**DOI:** 10.5937/jomb0-52348

**Published:** 2025-06-13

**Authors:** Arikan Funda Bulut, Nevin Sagsoz

**Affiliations:** 1 Kirikkale University, Faculty of Medicine, Department of Physiology, Kirikkale, Turkey; 2 Kirikkale University, Faculty of Medicine, Department of Obstetrics and Gynecology, Kirikkale, Turkey

**Keywords:** matriptase-2, neurokinin B, menopause, obesity, matriptaza-2, neurokinin B, menopauza, gojaznost

## Abstract

**Background:**

Matriptase-2 (MT2) is implicated in iron metabolism, obesity, insulin resistance, and glucose homeostasis. Neurokinin B (NKB) plays a crucial role in gonadotropin secretion, which is essential for reproduction. The impact of menopause and obesity on MT2 and NKB concentrations remains an enigma. Therefore, the present study sought to investigate how obesity and menopause affect serum concentrations of MT2 and NKB.

**Methods:**

In this cross-sectional study, 120 female volunteers were divided into four groups: menopausal overweight, menopausal obese, menopausal normal weight (control), and reproductive normal weight (control). The participants' serum levels of MT2, NKB, iron, ferritin, and relevant biochemical parameters were analysed using the ELISA method.

**Results:**

The mean serum concentrations of NKB, MT2, and ferritin were higher in the menopausal overweight and obese groups (p<0.05). The mean concentrations of NKB, MT2, and estradiol were determined to be higher, and the levels of iron, hemoglobin, glucose, FSH, LH, LDL, and total cholesterol were found to be lower in the reproductive control group when compared with the menopausal control group (p<0.05). A positive correlation was identified between BMI and NKB, MT2, and ferritin concentrations in menopausal overweight and obese groups (p<0.05).

**Conclusions:**

Obesity elevated NKB and MT2 concentrations in menopausal women. The increase in MT2 levels in menopausal overweight or obese women may be one of the factors responsible for the increase in ferritin and body fat ratio. As BMI raised, NKB, MT2, and ferritin concentrations raised in the menopausal overweight or obese groups; therefore, depression of MT2 in obese patients could be beneficial for therapeutic purposes. Additionally, gonadal hormonal changes altered serum concentrations of NKB, and the increase in NKB and gonadotropin levels in menopause may enhance vasomotor symptoms.

## Abstract (sr)

**Uvod:**

Matriptaza-2 (MT2) učestvuje u metabolizmu gvožđa, gojaznosti, insulinskoj rezistenciji i homeostazi glukoze. Neurokinin B (NKB) ima ključnu ulogu u sekreciji gonadotropina, koji su neophodni za reprodukciju. Uticaj menopauze i gojaznosti na koncentracije MT2 i NKB ostaje nejasan. Cilj ove studije je bio da istraži kako gojaznost i menopauza utiču na serumske koncentracije MT2 i NKB.

**Metode:**

U ovoj studiji preseka učestvovalo je 120 žena, podeljenih u četiri grupe: žene u menopauzi s prekomernom telesnom težinom, gojazne žene u menopauzi, žene u menopauzi s normalnom telesnom težinom (kontrolna grupa) i žene u reproduktivnom periodu s normalnom telesnom težinom (kontrolna grupa). Serumski nivoi MT2, NKB, gvožđa, feritina i relevantnih biohemijskih parametara analizirani su ELISA metodom.

**Rezultati:**

Prosečne serumske koncentracije NKB, MT2 i feritina bile su više u grupama žena u menopauzi s prekomernom telesnom težinom i gojaznošću (p<0,05). U grupi žena u reproduktivnom periodu kontrolne vrednosti NKB, MT2 i estradiola bile su više, dok su nivoi gvožđa, hemoglobina, glukoze, FSH, LH, LDL i ukupnog holesterola bili niži u poređenju sa kontrolnom grupom žena u menopauzi (p<0,05). Pozitivna korelacija identifikovana je između BMI i koncentracija NKB, MT2 i feritina u grupama žena u menopauzi s prekomernom težinom i gojaznošću (p<0,05).

**Zaključak:**

Gojaznost povećava koncentracije NKB i MT2 kod žena u menopauzi. Povećanje nivoa MT2 kod žena u menopauzi s prekomernom telesnom težinom ili gojaznošću može biti jedan od faktora odgovoran za povećanje nivoa feritina i procenta telesne masti. Kako se BMI povećava, povećavaju se i koncentracije NKB, MT2 i feritina u grupama žena u menopauzi sa prekomernom težinom ili gojaznošću, te bi smanjenje MT2 kod gojaznih pacijentkinja moglo da ima terapeutski značaj. Pored toga, hormonalne promene gonada utiču na serumske koncentracije NKB, a povećanje NKB i gonadotropina u menopauzi može doprineti jačim vazomotornim simptomima.

## Introduction

Menopause is a physiological process that results in the permanent cessation of menstruation caused by the loss of ovarian follicular activity due to age, chemotherapy, or surgical treatment [1]. Menopause is associated with increased visceral, abdominal, and subcutaneous fat and causes a significant increase in total body fat. In particular, abdominal fat accumulation is crucial in developing insulin resistance, type 2 diabetes, and cardiovascular diseases in women [2] [3] [4] [5] [6] [7] [8]. However, the pathophysiology of obesity in menopausal women is not fully understood. Hormonal changes in perimenopause are known to increase abdominal obesity. In particular, it is characterised by a sudden drop in estrogen levels and increased abdominal subcutaneous and visceral fat [9] [10]. Stubbins et al. [10] showed that estrogen protects female mice against adipocyte hypertrophy, adipose tissue oxidative stress, inflammation, liver steatosis, and insulin resistance. Matriptase-2 (MT2), which we investigated in this study, is a serine protease encoded by the transmembrane protease serine 6 (TMPRSS6) gene, and it is a key regulator of iron metabolism by repressing hepcidin expression [11]. Hepcidin inhibits iron transportation into the bloodstream due to it is direct binding to iron transporter ferroportin, which is localised in the cell membrane of duodenal enterocytes that reabsorbs dietary iron and macrophages that take up iron from senescent erythrocytes. Hepcidin induces ferroportin internalisation and breakdown in lysosomes by binding to ferroportin, thus blocking the flow of iron in the circulatory system [12]. Further, MT2 inhibits hepcidin expression and reduces the amount and activation of hepcidin by cleaving membrane hemo juvelin. Therefore, high expression levels of MT2 suppress hepcidin synthesis, resulting in increased plasma iron [11] [13].

During menopause, women experience considerable changes in their iron and ferritin levels. Postmenopausal women have an increase in iron and iron stores compared with premenopausal women. The main reason for this increase in iron is the cessation of menstrual bleeding. Reduced estrogen levels in menopause may cause increases in total fat mass or obesity and influence iron status [14] [15]. However, researchers do not know whether other factors also contribute to this increase. This study aimed to investigate whether there is a relationship between menopausal changes in iron and ferritin concen trations and matriptase-2 (MT2). Furthermore, researchers have recently stated that MT2 plays a profound role in obesity, insulin resistance, and glucose homeostasis [16], although the effects of MT2 on obesity remain unclear. To clarify this uncertainty, the present study examined the circulating levels of MT2 in overweight or obese menopausal women.

Another factor examined in the present study is neurokinin B (NKB), encoded by the tachykinin precursor 3 (TAC3) gene. Along with kisspeptin, NKB is an essential modulator of GnRH and, thus, gonadotropin secretion. Therefore, NKB is crucial for puberty and reproductive function [17] [18]. Menopause-induced hypertrophy of neurons in the arcuate nucleus of the hypothalamus in postmenopausal women and increased expressions of kisspeptin, NKB, and dynorphin (KNDy) are a compensatory response to ovarian failure [17] [18]. Arcuate nucleus neurons that express KNDy have a crucial role in sex-steroid feedback on gonadotropin secretion in humans, which suggests that these arcuate nucleus neurons may be part of the neural network responsible for increased serum levels of gonadotropin in postmenopausal women [18]. Researchers have suggested that NKB may also be responsible for the change in gonadotropin levels in menopausal women.

Therefore, this study examines the circulatory levels of NKB in menopausal women to detect decreases or increases.

## Materials and methods

### Subjects

All volunteers registered at the gynecology and obstetrics clinic of Kirikkale University Medical Faculty Hospital were informed in detail regarding the study, and written informed consent was received from each participant. All required permissions were obtained from Kirikkale University clinical research ethics committee for the present study. This study was performed in line with the principles of the Declaration of Helsinki.

This study involved 120 female volunteers divided evenly into four groups: menopausal overweight, menopausal obese, menopausal normal weight (control), and reproductive normal weight (control). Serum levels of MT2, NKB, and relevant biochemical parameters were analysed.

Exclusion criteria for menopausal volunteers: potential participants were excluded if they had diseases such as AIDS, cancer, endocrine disorders (such as diabetes mellitus or thyroid disorder), hepatitis, or tuberculosis, among others. The use of hormone drugs in the past year was another disqualifier. Women were also excluded if they were experiencing any form of induced menopause, such as that resulting from bilateral ovariectomy (OVX) or radiation therapy.

Inclusion criteria for menopausal volunteers: potential participants did not have a menstrual period for at least the past 12 months and did not have any of the exclusion criteria.

Exclusion criteria for reproductive control: potential participants were excluded if they had diseases such as AIDS, cancer, endocrine disorders (such as diabetes mellitus or thyroid disorder), hepatitis, or tuberculosis, among others. The use of hormone drugs in the past year was another disqualifier.

Inclusion criteria for reproductive control: having a regular menstrual pattern and not meeting any exclusion criteria.

### Study protocol

Participants’ specific clinical and demographic characteristics, such as vasomotor symptom status, menstrual patterns, age, height, and weight, were recorded. BMI [weight (kg)/height (m)^2^] was calculated. BMI was classified as normal (BMI 18.5–24.9), overweight (BMI 25–29.9), or obese (BMI³30.0).

Venous blood samples were drawn from a forearm in the morning after eight hours of fasting. Blood samples were centrifuged to obtain serum within 30 minutes of blood draws. Serum samples were kept at -80°C until serum levels of MT2, NKB, ferritin, iron, hemoglobin, hematocrit, glucose, insulin, FSH, LH, estradiol, and lipid panels were analysed. Hemoglobin and hemogram values were measured with the blood placed into EDTA tubes. The homeostatic model assessment of insulin resistance index (HOMA-IR) was used to quantify insulin resistance = Fasting glucose (mg/dL) x Fasting serum insulin (μU/mL)/405.

Serum levels of estradiol, FSH, LH, and insulin were detected using the electrochemiluminescence immunoassay technique with the Cobas 8000 analyser and Roche Cobas kits (Roche Diagnostics GmbH, Mannheim, Germany). Serum lipid panels and glucose and iron levels were measured with the Mindray chemistry analyser BS-2000M and a Mindray reagent kit based on colourimetric methodology (Mindray Bio-Medical Electronics Co., Hamburg, Germany).

The NKB serum concentrations were determined using an NKB enzyme-linked immunosorbent assay (ELISA) kit (Phoenix Pharmaceuticals, Inc., Burlingame, CA, USA. Catalog no.: EK-046-26; range: 0–100 ng/ml), and MT2 serum concentrations were determined using human TMPRSS6 and an ELISA Kit (Abbexa Ltd., Cambridge, UK. Catalog no.: abx573612; range: 0.312 ng/mL–20 ng/mL). Ferritin serum concentrations were detected using a human DiaMetra ELISA Kit (DiaMetra Ltd., Segrate, Italy). The absorbance measurements of optical densities were detected at a wavelength of 450 nm by placing the immuno plates in a microplate reader (BioTek Instruments, Winooski, VT, USA) during the final stage of each ELISA.

### Statistical analysis

The statistical analyses were conducted using SPSS, version 20.0 (IBM SPSS Statistics, Armonk, NY, USA), and the significance level was set at p<0.05. Shapiro-Wilk test was used to assess the normality analysis. Differences among the means of data in the menopausal over-weight, menopausal obese, and menopausal control groups were determined using a one-way ANOVA followed by post-hoc Tukey’s test. All parameters in the menopausal and reproductive control groups were determined using an independent samples t-test.

The correlation between parameters was evaluated using Pearson or Spearman two-way correlation tests.

## Results

**Figure 1:**
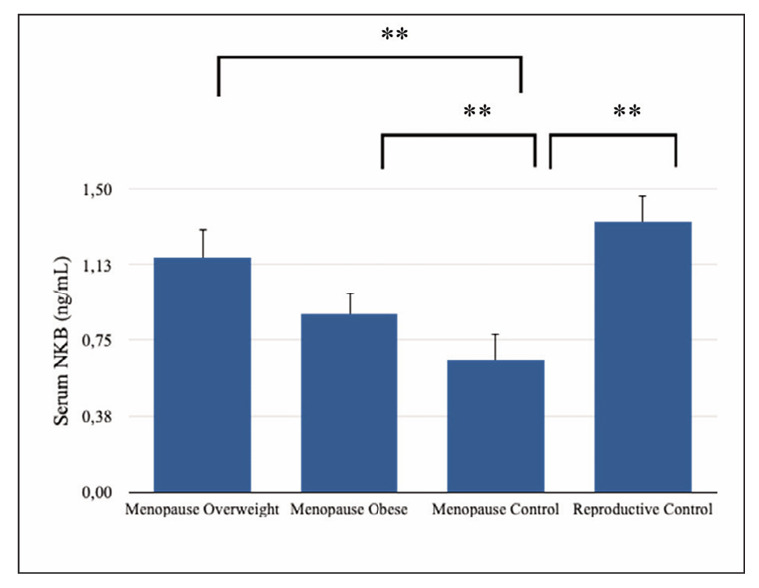
Serum neurokinin B concentrations. Data shown are mean ± STD. *P<0.05, **P=0.000, posthoc Tukey’s test and Independent samples t-test. NKB: Neurokinin B

**Figure 2:**
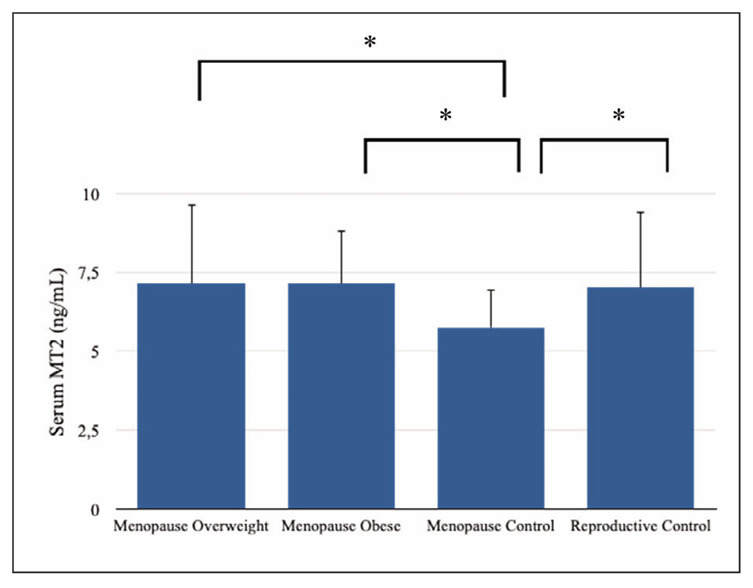
Serum matriptase-2 concentrations. Data shown are mean ± STD. *P<0.05, **P=0.000, posthoc Tukey’s test and Independent samples t-test. MT2: Matriptase-2

Anthropometric and clinical data for the groups are shown in Table 1. The mean serum levels of MT2 and NKB were statistically significant among meno pausal overweight, menopausal obese, menopausal control, and reproductive control groups (p<0.05, Table 1, Figure 1 and Figure 2). The mean serum levels of NKB, MT2, and ferritin were higher in the menopausal overweight and obese groups than in the menopausal control group (p<0.05).

**Table 1:** Comparison of anthropometric and clinical data. Menopause overweight and obese groups were compared with the menopause control by post-hoc Tukey’s test. Menopause control was compared with reproductive control by independent samples of the t-test. Data shown are mean ± STD. *P<0.05, **P=0.000.

	Menopause<br>Overweight		Menopause<br>Obese		Menopause<br>Control	Reproductive<br>Control	
	Mean ± STD	p	Mean ± STD	p	Mean ± STD	Mean ± STD	p
Neurokinin B (ng/mL)	1.16±0.14	0.000**	0.88±0.10	0.000**	0.65±0.13	1.34±0.11	0.000**
Matriptase-2 (ng/mL)	7.14±2.5	0.009*	7.13±1.7	0.001*	5.76±1.2	7.03±2.4	0.008*
Age	55±5.7	0.37	56±5.4	0.15	54±5.5	25.2±0.09	0.000**
BMI	28.4±1.4	0.000**	32.1±1.5	0.000**	23.2±1.7	22.3±2.2	0.10
Iron (ug/dL)	84.4±34	0.91	84.9±35	0.92	88.9±37	63.6±35.1	0.04*
Ferritin (μg/mL)	77.8±60	0.02*	129.9±53	0.000**	43.5±37	36.8±13.1	0.64
Hemoglobin (g/dL)	13.6±0.97	0.52	13.9±0.92	0.85	13.8±1.3	13.0±1.1	0.02*
Hematocrit (%)	41±2.9	0.28	43±4.0	0.72	42±3.3	40.9±2.8	0.09
Glucose (mg/dL)	96.7±11.6	0.78	99.8±12	0.43	97.5±9.5	89.2±10.2	0.008*
Insulin (μU/mL)	8.2±2.1	0.77	11.4±4.9	0.22	7.7±3.0	10.6±4.2	0.26
FSH (mIU/mL)	48.4±25.2	0.86	54.9±28.6	0.43	46.8±17.4	6.5±1.4	0.000**
LH (mIU/mL)	30.3±12.8	0.56	36.6±18.9	0.79	34.4±22.9	7.4±5.6	0.000**
Estradiol (pg/mL)	15.9±9	0.32	12.3±10.2	0.06	19.8±12.7	39.07±20.8	0.001*
Triglyceride (mg/dL)	127.8±46.4	0.45	144.9±47.4	0.10	114.8±55.1	104.9±45.2	0.61
LDL (mg/dL)	123.7±33.2	0.26	124.5±23.9	0.15	112±24.3	93.4±21.9	0.04*
HDL (mg/dL)	61±15.2	0.75	58±13.5	0.42	63±19.4	59.2±115	0.55
Total Cholesterol (mg/dL)	211±15.1	0.05	206±31.9	0.44	197±28.1	174.4±22.2	0.02*

The mean serum levels of NKB, MT2, and estradiol were higher in the reproductive control group, whereas the mean serum levels of iron, hemoglobin, glucose, FSH, LH, LDL, and total cholesterol were lower in the reproductive control group than in the menopausal control group (p<0.05, Table 1).

**Figure 3:**
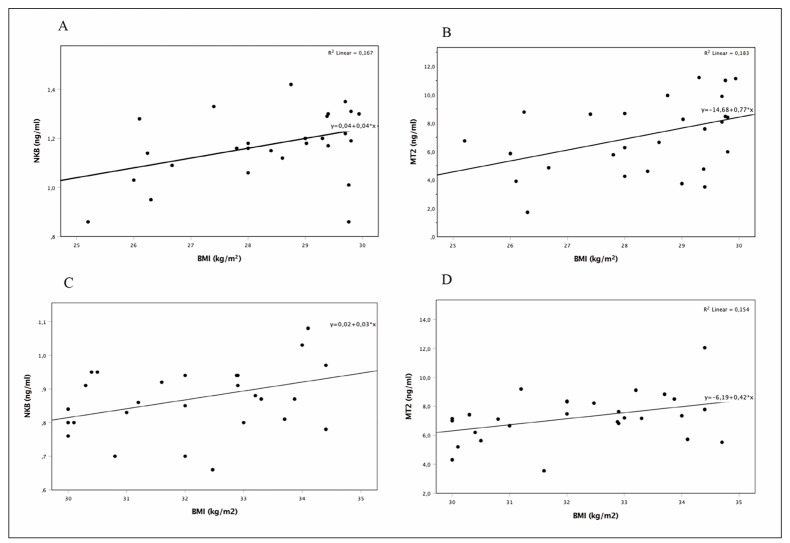
Correlation analyzes. A, B; Serum NKB and MT2 correlations with BMI in menopause overweight group. C, D; Serum NKB and MT2 correlations with BMI in menopause obese group. NKB: Neurokinin B, MT2: Matriptase-2

Correlation analyses were also performed, and the results are presented in Table 2. Serum NKB and MT2 correlations with BMI in the menopausal overweight and obese groups are presented in Figure 3A, Figure 3B, Figure 3C, and Figure 3D.

**Table 2:** Correlation analysis. *P*<0.05, **P=0.000.

	Menopause Overweight				Menopause Obese				Menopause Control			
	Neurokinin B		Matriptase-2		Neurokinin B		Matriptase-2		Neurokinin B		Matriptase-2	
	r	p	r	p	r	p	r	p	r	p	r	p
Age	0.054	0.78	0.077	0.69	0.111	0.58	0.007	0.97	-0.039	0.83	-0.218	0.24
BMI	0.409*	0.03	0.428*	0.02	0.387*	0.04	0.392*	0.03	-0.047	0.80	-0.097	0.61
Iron	0.129	0.58	0.595**	0.006	-0.297	0.12	0.642**	0.000	-0.244	0.20	0.748**	0.000
Ferritin	0.003	0.98	0.490**	0.007	-0.025	0.90	0.608**	0.001	0.017	0.92	0.441*	0.01
Hemoglobin	0.391*	0.03	0.382*	0.04	-0.025	0.89	0.394*	0.03	-0.044	0.81	0.105	0.58
Hematocrit	0.095	0.64	0.074	0.72	0.205	0.29	0.202	0.30	-0.076	0.68	0.143	0.45
Glucose	0.454*	0.01	0.458*	0.01	-0.337	0.08	0.509**	0.007	-0.263	0.21	-0.075	0.72
Insulin	0.439	0.23	0.299	0.43	0.092	0.70	0.360	0.11	0.643	0.55	-0.506	0.66
FSH	0.216	0.40	0.210	0.41	0.594*	0.02	-0.197	0.48	-0.388	0.26	0.223	0.53
LH	0.529*	0.04	-0.160	0.56	0.195	0.50	-0.447	0.10	-0.544	0.08	-0.047	0.89
Estradiol	-0.231	0.38	0.008	0.97	0.504	0.56	0.293	0.27	0.021	0.93	-0.189	0.43
Triglyceride	0.079	0.73	0.078	0.73	0.200	0.42	0.051	0.84	-0.116	0.69	0.241	0.40
LDL	0.169	0.46	-0.085	0.71	0.435	0.07	-0.517*	0.02	-0.238	0.41	0.136	0.64
HDL	0.286	0.18	-0.122	0.57	-0.190	0.44	0.007	0.97	-0.066	0.82	-0.334	0.24
Total Cholesterol	0.318	0.14	-0.007	0.97	-0.064	0.80	0.086	0.73	-0.293	0.30	0.082	0.78
Vasomotor symptom	0.139	0.49	0.228	0.25	0.436*	0.03	0.242	0.25	-0.270	0.17	0.161	0.42

In the menopausal overweight group, the MT2 concentration showed a statistically significant positive correlation with BMI as well as levels of hemoglobin, iron, ferritin, and glucose, and the NKB concentration exhibited a positive correlation with BMI, hemoglobin, glucose, and LH (*p*<0.05, Table 2). Furthermore, in the menopausal overweight group, a positive correlation was identified between BMI and ferritin, glucose, and hemoglobin levels (*r*=0.393, *p*=0.03; *r*=0.559, *p*=0.003; and *r*=0.513, *p*=0.005, respectively).

In the menopausal obese group, a positive correlation was identified between the MT2 concentration and BMI and levels of hemoglobin, iron, ferritin, and glucose, while a negative correlation was found between the MT2 concentration and the LDL level. A positive correlation was also observed between the NKB concentration and BMI, FSH, and vasomotor symptoms. A positive correlation was detected between BMI and ferritin levels (*r*=0.459, *p*=0.016), while a negative correlation was noted between vasomotor symptoms and LH (*r*=-0.657, *p*=0.015).

In the menopausal control group, a positive correlation was found between the MT2 concentration and iron and ferritin levels (*p*<0.05, Table 2).

In the reproductive control group, the MT2 concentration exhibited statistically significant positive correlations with age, iron and ferritin levels (*r*=0.505, *p*=0.006; *r*=0.694, *p*=0.001; and *r*=0.517, *p*=0.033, respectively). The NKB concentration was positively correlated with LH level (*r*=0.521, *p*=0.015). A negative correlation was found between BMI and HDL levels (*r* =-0.501, *p*=0.04).

## Discussion

The present study represents the first time that menopause has been found to cause a significant decrease in serum levels of NKB. However, MT2 concentrations were higher in menopausal overweight, menopausal obese, and reproductive control groups compared with the menopausal control group, while obesity increased the serum concentrations of NKB and MT2 in menopausal women. The relationship of MT2 to the pathophysiology of obesity is not yet known. Only one recent animal study indicates that the absence of the MT2 gene protects against obesity caused by high-fat diets. Researchers have determined that a significant decrease in hepatic steatosis, body fat, and plasma triglycerides improves glucose tolerance, insulin sensitivity, and enhanced fat lipolysis in TMPRSS6−/− mice, which provides information regarding the importance of MT2 and iron regulation in obesity [16] [19]. In light of this animal study, the increase in MT2 levels in the present study’s obese women may be one of the factors responsible for the rise in the obese body fat ratio.

Additionally, the detection in this study of a significant positive correlation between MT2 concentrations and BMI, as well as glucose levels in menopausal overweight and obese groups, is supported by the animal study results. The cause-and-effect relationship between MT2 and human obesity has not yet been clarified. If further studies confirm such a relationship, this information may guide treatment approaches in the future. In the present study, ferritin levels were statistically significantly higher in the menopausal overweight and obese groups than in the menopausal control group, and a statistically significant positive correlation was noted between BMI and ferritin levels in the menopausal overweight and obese groups. Aprior study emphasises that hepcidin production increases in patients with increased obesity [20]. Thus, the high ferritin levels in the overweight and obese groups in the present study can result from repressed hepcidin levels due to the rise in MT2 concentrations. The results of the present study suggest that as BMI increases in menopausal women, ferritin concentration also rises. Ferritin is an acute-phase reactant that increases in the presence of infection and inflammation; therefore, ferritin is an inflammation marker. Excess adipose tissue in obese patients causes inflammation and increased ferritin levels [21] [22].

Furthermore, in the current study, the serum levels of iron in the menopausal control group were statistically higher than in the reproductive control group. The primary reason for this increase is the cessation of menstrual bleeding. Moreover, the decline in estrogen in menopause can lead to increased total fat mass or obesity and may affect iron status [14] [15].

According to the results of the present study, increased levels of MT2 in the menopausal overweight or obese groups may be responsible for the elevated ferritin levels. Detecting a positive correlation between MT2 and ferritin in these groups supports this result. Furthermore, the results of a previous study of mice with a TMPRSS6 (MT2) gene knockout in their retinas revealed downregulation of ferritin levels, which indicates that MT2 deficiency could lead to ferritin deficiency [23].

The role of MT2 in fertilisation is not yet known, but no correlation was determined in this study between MT2 concentration and FSH, LH, and estradiol levels in the menopausal groups. Since MT2 is also synthesised from the ovaries [24], MT2 concentrations may have decreased in the menopausal control group compared to the reproductive control group.

In the present study, the researcher determined that obesity elevated the NKB concentrations in menopausal women. However, NKB levels were higher in the reproductive control group compared to the menopausal control group. A high-fat diet and type 2 diabetes study conducted with rats by Ziarniak et al. [25] demonstrated that the number of NKB neurons was higher in post-OVX hormone replacement with estradiol and post-OVX hormone replacement with estradiol and progesterone than in an OVX group. The low NKB neuron count in OVX rats without hormone replacement aligns with the higher NKB concentration in the reproductive control group compared to the menopausal control group in this study. The results of the current study reveal that gonadal hormonal changes between the reproductive and menopausal control groups altered the serum concentrations of NKB regardless of obesity.

Ziarniak et al. [25] further suggest that meta bolic changes, such as a high-fat diet and diabetes mellitus, affect the number of NKB cells in the arcuate nucleus.

The present study reveals for the first time that obesity elevates NKB concentrations in menopausal women. Furthermore, the detection of a positive correlation between BMI and NKB in menopausal overweight and obese groups supports this conclusion. However, a prior study indicates that KNDy neuron ablation blocks the effects of OVX and E2 replacement on body weight and abdominal girth in rats. Compared with the control group, E2 treatment did not cause weight loss or changes in abdominal circumference in KNDy-ablated rats, and there was no significant weight gain in these rats three weeks after OVX. Consequently, the study revealed that KNDy neurons are crucial to gonadotropin secretion, the increase of LH after removal of E2, and the E2 regulation of body weight [26]. In an animal study, neurokinin receptor agonists were shown to reduce body weight and blood glucose levels in obese animals, with a more pronounced effect observed in diabetic subjects.

Furthermore, these agonists decreased food intake and improved insulin resistance. These findings underscore the potential therapeutic application of these agents in treating obesity [27]. In this study, it was found that neurokinin B levels were elevated in obese women. Considering the relevant animal study, this increase may represent a compensatory mechanism to mitigate the adverse effects of obesity.

According to the results of the present study, a positive correlation was noted between the NKB concentration and vasomotor symptoms in the menopausal obese group. Prior studies have indicated that NKB is associated with vasomotor symptoms, such as hot flashes and night sweats; their aetiology remains unknown. Consequently, researchers have suggested that NKB antagonists can effectively treat vasomotor symptoms [26] [28] [29] [30]. A negative correlation was noted between vasomotor symptoms and LH in the menopausal obese group in the present study. Therefore, the increase in NKB and gonado tropin levels in menopause may exacerbate vasomotor symptoms, although more studies are needed on this subject.

## Conclusions

The role of obesity and menopausal changes in NKB and MT2 levels and iron and ferritin metabolism remains unclear. The results of the present study indicate that the increase in BMI in overweight or obese menopausal women increases NKB, MT2 and ferritin concentrations. Increased levels of MT2 may be responsible for the elevated ferritin levels. Serum concentrations of NKB and MT2 were higher in the reproductive control group than in the menopausal control group. Gonadal hormonal changes between the two control groups altered serum concentrations of NKB. To the researcher’s knowledge, the present study represents the first time obesity has been determined to elevate NKB and MT2 serum concentrations in menopausal women. The increase in MT2 levels may be one of the factors responsible for the rise in the body fat ratio in obese women; therefore, depression of MT2 concentrations in obese female patients can be used clinically for therapeutic purposes. The increase in NKB and gonadotropin levels in menopause may exacerbate vasomotor symptoms, although more studies are needed on this subject.

## Dodatak

### Author contributions

FBA participated in the development of the study concept and design, performing the laboratory analysis, the analysis of the data, the acquisition or interpretation of data and results, and the drafting of the manuscript.

NS participated in the development of the study concept and design, the analysis of the data, the acquisition or interpretation of data and results, and the drafting of the manuscript.

All authors read and approved the final manuscript; no other person contributed substantially to the paper.

### Ethics statement

All volunteers registered at the gynecology and obstetrics clinic of Kirikkale University Medical Faculty Hospital were informed in detail regarding the study, and written informed consent was received from each participant. All required permissions were obtained from Kirikkale University clinical research ethics committee for the present study. (Number of meetings: 2019/02, meeting decision number: 02/07). This study was performed in line with the principles of the Declaration of Helsinki.

There is no reproduced material from other sources.

This study was supported by the Scientific Research Projects Coordination Unit of Kirikkale University (Project number: 2019/055).

### Conflict of interest statement

All the authors declare that they have no conflict of interest in this work.
